# Role of extracellular matrix components in biofilm formation and adaptation of *Pseudomonas ogarae* F113 to the rhizosphere environment

**DOI:** 10.3389/fmicb.2024.1341728

**Published:** 2024-01-25

**Authors:** Esther Blanco-Romero, Daniel Garrido-Sanz, David Durán, Morten Rybtke, Tim Tolker-Nielsen, Miguel Redondo-Nieto, Rafael Rivilla, Marta Martín

**Affiliations:** ^1^Departamento de Biología, Facultad de Ciencias, Universidad Autónoma de Madrid, Madrid, Spain; ^2^Department of Fundamental Microbiology, University of Lausanne, Lausanne, Switzerland; ^3^Costerton Biofilm Center, Department of Immunology and Microbiology, University of Copenhagen, Copenhagen, Denmark

**Keywords:** biofilm, *Pseudomonas ogarae* F113, extracellular matrix components, rhizosphere, surface attachment, motility, flow cell, dynamic biofilm formation

## Abstract

Regulating the transition of bacteria from motile to sessile lifestyles is crucial for their ability to compete effectively in the rhizosphere environment. *Pseudomonas* are known to rely on extracellular matrix (ECM) components for microcolony and biofilm formation, allowing them to adapt to a sessile lifestyle. *Pseudomonas ogarae* F113 possesses eight gene clusters responsible for the production of ECM components. These gene clusters are tightly regulated by AmrZ, a major transcriptional regulator that influences the cellular levels of c-di-GMP. The AmrZ-mediated transcriptional regulation of ECM components is primarily mediated by the signaling molecule c-di-GMP and the flagella master regulator FleQ. To investigate the functional role of these ECM components in *P. ogarae* F113, we performed phenotypic analyses using mutants in genes encoding these ECM components. These analyses included assessments of colony morphology, dye-staining, static attachment to abiotic surfaces, dynamic biofilm formation on abiotic surfaces, swimming motility, and competitive colonization assays of the rhizosphere. Our results revealed that alginate and PNAG polysaccharides, along with PsmE and the fimbrial low molecular weight protein/tight adherence (Flp/Tad) pilus, are the major ECM components contributing to biofilm formation. Additionally, we found that the majority of these components and MapA are needed for a competitive colonization of the rhizosphere in *P. ogarae* F113.

## 1 Introduction

The rhizosphere is a highly competitive and complex environment, where the survival of rhizobacteria depends on their ability to respond to changes and to switch between planktonic and sessile lifestyles. Sessility is frequently associated with the protective strategy of biofilm formation where bacteria are found in surface-associated communities (Costerton et al., 1995), typically embedded in a self-produced extracellular matrix (ECM). This strategy enhances the ability of bacteria to cope and survive in harsh environmental conditions (Stewart and Costerton, 2001; Hall-Stoodley et al., 2004) and establish interactions with hosts (Parsek and Fuqua, 2004). Indeed, plant-associated bacteria can attach and form biofilms or microcolonies on plant surfaces, which constitutes an adaptive strategy for the successful colonization (Solanki et al., 2020). In *Pseudomonas*, a wide range of polysaccharides and extracellular proteins have been described as key components of ECMs (Blanco-Romero et al., 2022b). Polysaccharides serve to anchor bacterial cells to surfaces, provide structure to biofilms, maintain optimal humidity levels, and protect organisms from harmful molecules. They also act as host-colonization factors to prevent or delay host defense activation responses (Denny, 1995; Leid et al., 2005). Therefore, the production of polysaccharides has been widely studied in pathogenic *Pseudomonas* species, such as the human pathogen *P. aeruginosa* (Ramphal and Pier, 1985; Fazli et al., 2014; Jones and Wozniak, 2017; Harrison et al., 2020; Rybtke et al., 2020), the insect pathogen *P. protegens* (Vesga et al., 2020), and the phytopathogen *P. syringae* (Fett et al., 1989; de Pinto et al., 2003; Arrebola et al., 2015; Heredia-Ponce et al., 2020a). Furthermore, polysaccharides have also been studied in commensal *Pseudomonas* species (Gal et al., 2003; Ude et al., 2006; Nielsen et al., 2011; Nilsson et al., 2011; Heredia-Ponce et al., 2020b).

Aside from polysaccharides, other major components of ECMs are extracellular proteins, such as adhesins, the type IV pili and functional amyloids. Adhesins are proteins expressed on the bacterial surface and are important for attachment to both abiotic and biotic surfaces. Adhesins are crucial for bacteria-host interaction as described for the large adhesion protein A (LapA) in *P. fluorescens* and *P. putida*, which mediates colonization and attachment to the plant (Espinosa-Urgel et al., 2000; Hinsa et al., 2003; Yousef-Coronado et al., 2008). Another adhesin, the medium adhesion protein (MapA), has been shown to play a role in biofilm formation in *P. fluorescens* (Collins et al., 2020). The type IV pili forms surface appendages considered part of the ECM that are also involved in biofilm formation, colonization, and adhesion to host cells, among other functions (Lugtenberg et al., 2001; Cole et al., 2017). Functional amyloids are also crucial in bacteria-plant interaction. These include TasA, which mediates *Bacillus subtilis* biofilm formation on plant leaves (Camara-Almiron et al., 2020), or Curli, which is needed for *Escherichia coli*-plant interaction (Fink et al., 2012; Carter et al., 2016), In *Pseudomonas fluorescens* Pf07 the functional amyloid Fap, which has been described in *Pseudomonas fluorescens* Pf07 as the main contributor to the formation of a mature multicellular biofilm (Dueholm et al., 2013; Zeng et al., 2015; Guo et al., 2022). In addition, other structures such as the TasA fibers characterized in *Bacillus subtilis*, contribute the formation of biofilms on plant leaves (Camara-Almiron et al., 2020) However, more data about the role of diverse ECMs in commensal *Pseudomonas*-plant interactions are needed to elucidate the implication of each component in the process of colonization and persistence on the plant tissues.

*Pseudomonas ogarae* F113, formerly known as *P. fluorescens* F113 (Garrido-Sanz et al., 2021), is a model bacterium for the study of competitive rhizosphere colonization (Capdevila et al., 2004; Barahona et al., 2010; Duran et al., 2021; Blanco-Romero et al., 2023; Durán et al., 2023). In this bacterium, the node formed by the two transcriptional factors (TF) AmrZ/FleQ is crucial for its adaptation to the rhizosphere environment (Martinez-Granero et al., 2014; Blanco-Romero et al., 2018; Muriel et al., 2018). The F113 genome harbors several gene clusters related to the synthesis of ECM components, most of which are subjected to regulation by AmrZ: the polysaccharides alginate, poly N-acetylglucosamine (PNAG), and Pseudomonas acidic polysaccharide (Pap), the extracellular proteins or proteinaceous structures functional amyloids in *Pseudomonas* (Fap), fimbrial low-molecular-weight protein/tight adherence (Flp/Tad) pilus, and mannuronan C-5 epimerase PsmE, and two adhesins LapA and MapA (Blanco-Romero et al., 2020). These genetic clusters are found conserved within phylogenetically related bacteria (Blanco-Romero et al., 2020). Although in some cases the transcriptional regulation of these components by AmrZ occurs directly, more commonly their regulation occurs indirectly through the second messenger c-di-GMP and/or FleQ (Martinez-Granero et al., 2014; Blanco-Romero et al., 2018; Muriel et al., 2018). The role and the functional importance of these ECM components in *P. ogarae* F113 biofilm formation, motility, and rhizosphere colonization have not yet been analyzed. In this work, we studied the contribution of each of the ECM components regulated by AmrZ in biofilm formation, motility and rhizosphere colonization of *P. ogarae* F113, showing that many of them are crucial for the adaptation of this bacterium to these environments.

## 2 Materials and methods

### 2.1 Bacterial strains, growth conditions, and antibiotics

Bacterial derivatives used in this work are listed in Supplementary Table S1. *Pseudomonas ogarae* F113 strains were routinely grown in LB (Bertani, 1951), SA (Scher and Baker, 1982), YMB (Vincent, 1970), Colonization Factor Antigen (CFA) (Dueholm et al., 2013) or Super Optimal broth with Catabolite repression (SOC) (Hanahan, 1983) media at 28°C. *E. coli* strains were grown in LB at 37°C. Growth medium and conditions specific for each experiment are detailed below. Antibiotics or dyes were added when needed to the following concentrations: kanamycin (Km), 25 or 50 μg/mL for *E. coli* and F113 respectively; gentamycin (Gm), 10 or 3 μg/mL for *E. coli* or F113; chloramphenicol (Cm), 30 μg/mL for *E. coli*; rifampicin (Rif), 100 μg/mL for F113, ampicillin (Amp), 100 μg/mL for *E. coli*; cycloheximide (Chx), 10 μg/mL; and 5-bromo-4-chloro-3-indolyl-β-D-galactopyranoside (X-gal), 40 μg/mL.

### 2.2 Mutant construction

*Pseudomonas ogarae* F113 mutants in the different extracellular matrix components were done by insertional inactivation. Regions of 100–1,000 bp of each gene were PCR-amplified (primers listed in Supplementary Table S2) and cloned into a pCR™2.1-TOPO^®^ vector (Invitrogen) in *E. coli* DH5α as the recipient strain. Subsequently, plasmid DNA was purified and spot-dialyzed in a membrane filter (Millipore VSWP02500) against distilled H_2_O. F113 electrocompetent cells were freshly prepared by washing with distilled water four times. Around 500 ng of the pCR™2.1-TOPO^®^ constructions were electroporated in 80 μL F113 competent cells for each mutant construction. Plasmid DNA-competent cell mix was incubated on ice for 2 min and then electroporated in a 0.1 cm gap cuvette (Bio-Rad) and a Gene Pulser Xcell Electroporation System (Bio-Rad) with the following settings: 1.8 kV, 50 μF, and 200 Ω. Immediately, cells were transferred to SOC medium and incubated for 2 h at 28°C with shaking at 220 rpm. Mutants were selected in SA plates with Km for 48 h at 28°C. Insertional mutants were checked by Southern Blot. Since the object of this mutagenesis was to eliminate the production of each of the extracellular matrix components and not to link particular genes with a function, possible polar effects were not taken into account and the presence of a phenotype was considered enough proof of lack of production of the ECM component.

For the exopolysaccharides Pap, PNAG, and alginate, the genes selected for disruption were those encoding the uridine diphosphate (UDP)-glucose/guanosine diphosphate (GDP) mannose dehydrogenase (*papA*), a porin (*pgaA*), and the polymerase (*alg8*), respectively. In the case of Fap, the selected genes were those encoding the minor nucleation protein (*fapB*), the fibril monomers (*fapC*) and the porin (*fapF*). For the mutant construction in the other extracellular proteins, the *mapA* and *psmE* genes were selected. Finally, for the Flp/Tad pilus, the gene encoding the major fibril component (*flp-1*) was chosen. The genetic organization of all these loci in *P. ogarae* F113 has been previously reported (Blanco-Romero et al., 2020). We have also analyzed mutants in genes encoding AmrZ and FleQ TFs because of their relevance in the environmental adaptation of *P. ogarae* F113 as both TFs regulate motility, ECM synthesis, and biofilm formation (Blanco-Romero et al., 2018).

### 2.3 Mini-Tn7-*gfp* strains construction

A mini-Tn7-*gfp* (Gm^R^) transposon cassette (Koch et al., 2001) was introduced into the chromosome of F113 wild-type and derivatives by four-parental mating using pRK600 (Finan et al., 1986) as coadjuvant and pUX-BF13 (Bao et al., 1991). GFP-tagged strains were selected in SA Gm-containing plates and checked in a GFP stereoscope Olympus SZX12.

### 2.4 Dyes staining

*Pseudomonas ogarae* F113 and derivatives were grown in YMB overnight at 28°C. 10 μL cultures were spotted in YMB agar 1.5% (w/v) plates supplemented with 100 μg/mL CR and grown at 28°C for 3 days. Images were taken for comparison on day three in a Leica MX125 stereoscope (zoom 1x25x).

### 2.5 Adhesion to surfaces

*Pseudomonas ogarae* F113 and derivatives were grown overnight in LB medium. Cultures were adjusted to an OD_600_ = 0.8, and 100 μL were inoculated in a 96-multiwell polystyrene treated plate. The multi-well plate was then incubated at 28°C statically for 2 h. Subsequently, media was removed and attached cells were fixed by the addition of 100 μL of 99% (v/v) methanol for 10 min, followed by air drying. Cells were stained with 100 μL of 1% (w/v) crystal violet solution for 15 min. Finally, excess staining was removed with distilled water by rising before dilution in 150 μL of a 33% (w/v) acetic acid solution for 15 min. The OD_590_ was measured using a microplate reader (BioTek Synergy HT). Experiments were repeated three times with 16 technical replicates per experiment.

### 2.6 Biofilm development in flow cells system

Overnight cultures of GFP-tagged *P. ogarae* F113 and derivatives were grown in LB medium. The OD_600_ was adjusted to 0.005 for the inoculation of the channels. Biofilms were grown at 25°C in three-channel flow cells (1 × 4 × 40 mm channel dimension) (Christensen et al., 1999) with a flow of 3 mL/h of minimal AB medium supplemented with 1 mM sodium citrate (Heydorn et al., 2000). The flow system was assembled and prepared as described by Christensen et al. (1999). The substratum consisted of a microscope glass coverslip (24 × 50 mm, thickness No. 1.5H; Marienfeld Laboratory Glassware). In each experiment, two separate channels were inoculated with each strain and two independent biofilm experiments were carried out. Strains were allowed to grow in the system for 48 h. For stability experiments, after 24 h growth, biofilms were challenged with 0.03% (w/v) SDS by switching the medium supply for 40 min.

### 2.7 Confocal laser scanning microscopy and image processing

Images were acquired using a Zeiss LSM 510 confocal laser scanning microscope (Carl Zeiss, Jena), 63x oil immersion objective, 488 nm argon laser line, and a 1.0 μm interval in the Z-stack. The number of slices was selected in each area to cover the biofilm completely. Six random Z-stacks, from the upper part of the channel at an approximately 5 mm distance from the inlet, were acquired per channel at 24 h post-inoculation for analysis, and two high-resolution images were taken for image processing. Additionally, two high-resolution images were acquired at 48 h post-inoculation for image processing and biofilm development comparison at later stages. For stability experiments, high-resolution images were taken at the same position before and after SDS-treatment. For image processing and data analysis, two images per channel for each derivative were captured before and after the treatment. The area occupied at substratum was calculated with COMSTAT2 (Heydorn et al., 2000; Vorregaard, 2008), using a fixed pixel background threshold of 15 with connected volume filtering as the filtering method.

Z-stack image processing was performed with Imaris 9.2.0 software (Bitplane): default background subtraction of 56.2 μm, section view, and different 3D-views (easy 3D with blend rendering and 3D-view option with shadow projection with maximum rendering quality and median filter 3x3x1). SDS experiment images were not subjected to background subtraction. Images were analyzed using COMSTAT2. Biofilms were quantified in terms of biomass and thickness-related traits, using an automatic Otsu’s thresholding method and the connected volume filtering method.

### 2.8 Swimming motility

*Pseudomonas ogarae* F113 and derivatives were grown overnight at 28°C in SA 1.5% (w/v) purified agar plates. Colonies were picked with a sterile toothpick and inoculated in the center of a 12 mL SA 0.3% (w/v) agar plate; 60 mm × 15 mm. Plates were grown at 28°C. Swimming motilities were measured as haloes diameters and images were taken at 24 h.

### 2.9 Competitive rhizosphere colonization assays

Alfalfa (*Medicago sativa* var. Resis) plants were used in competitive rhizosphere colonization assays. Seeds were surface-sterilized with 70% (v/v) ethanol, 5% (v/v) sodium hypochlorite, and washed gently with sterile distilled H_2_O. Seeds were then germinated in H_2_O 1% (w/v) purified agar for 48 h at 28°C. Seedlings were transferred to 50 mL tubes containing 25 mL of sterile pre-wetted medium-grain vermiculite and 10 mL FP solution (Fåhraeus, 1957) and grown in a controlled environment (16/8 h, light/dark photoperiod, and 25/18°C). One-week-old alfalfa plants were inoculated with a mixture of the control (F113-*gfp*) and competitor strain (ECM component mutant to be tested) at a 1:1 ratio and a OD_600_ equivalent to 1·10^3^ CFUs. Seven days after inoculation, shoots were removed, and bacteria present in the rhizosphere were resuspended in 20 mL of NaCl 0.75% (w/v) by vortex. Dilution series were platted in SA 1.5% (w/v) purified agar supplemented with Rif and Chx and grown for 72 h at 28°C. CFUs were plated onto fresh SA 1.5% (w/v) purified agar plates and grown for 24 h at 28°C. Control/competitor strains were distinguished by the presence/absence of GFP fluorescence in a GFP stereoscope (Olympus SZX12). Experiments were performed in triplicate with ten independent plants in each assay.

### 2.10 Statistical analysis

GraphPad Prism version 7.00 (GraphPad Software) was used in the statistical analysis and representation of swimming motility and biofilm formation assays using one-way analysis of variance (ANOVA) uncorrected Fisher’s least significant difference (LSD), and for COMSTAT2 data using two-tailed unpaired *t*-tests. COMSTAT2 data for stability experiments were analyzed with two-tailed *t*-tests without multiple comparison corrections.

## 3 Results

### 3.1 Polysaccharides and extracellular proteins contribute to biofilm formation in *Pseudomonas ogarae* F113

To study the role of AmrZ-regulated ECM components in the formation of biofilms by *P. ogarae* F113, we constructed mutants by insertional inactivation of genes affecting the production of each of these components.

**Figure 1:**
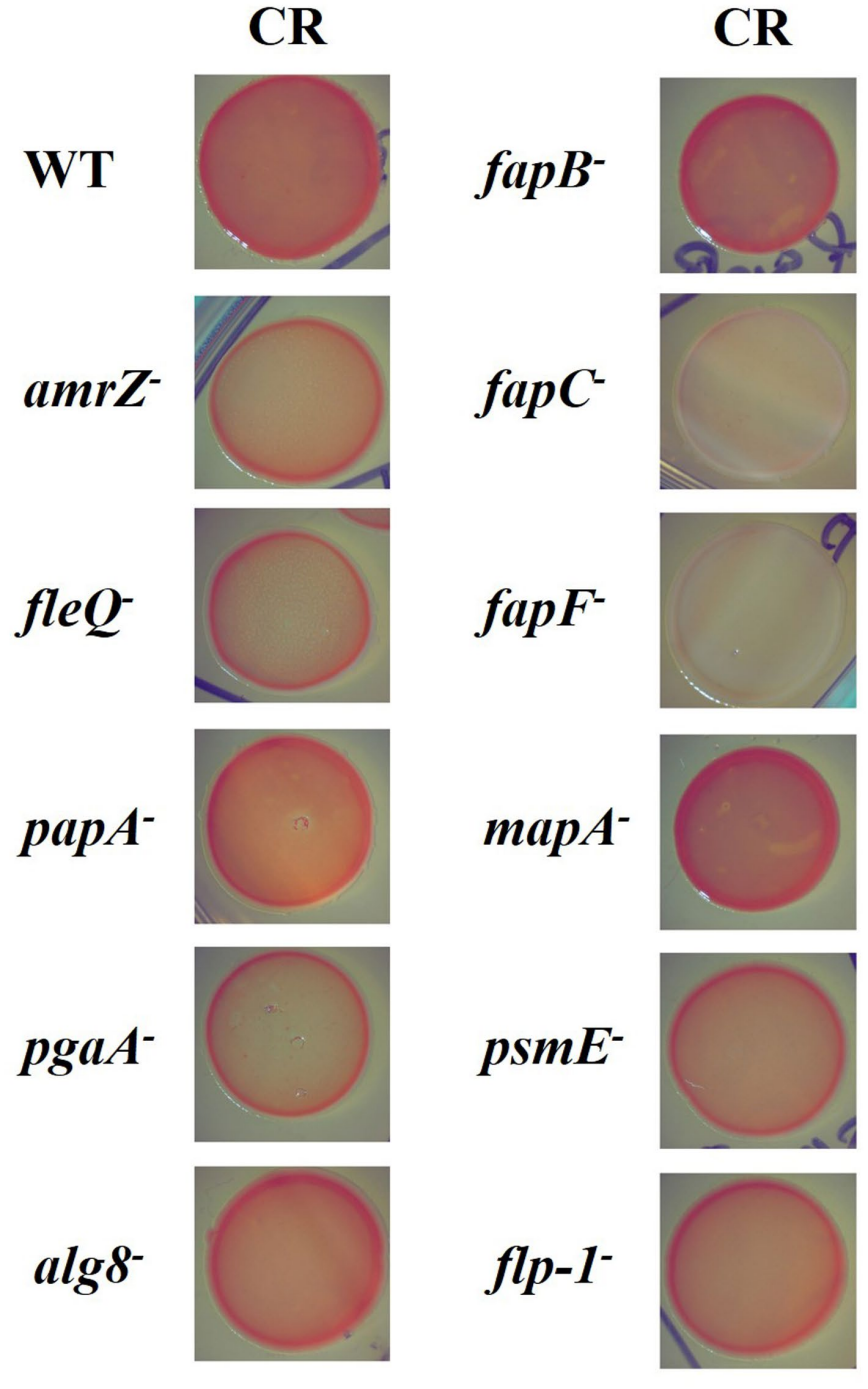
Dye-binding ability and colony morphology in *P. ogarae* F113 and mutants in extracellular matrix components. *P. ogarae* F113 wild-type (WT) and derivatives colonies growing in YMB plates supplemented with Congo Red (CR). Images depict colony growth after 3 days and are representative of at least three independent experiments with three replicates per experiment. Zoom x1.25.

The mutants affected in the production of ECM components were tested in macrocolony morphology and binding to Congo Red (CR) dye assay. CR is an unspecific dye that can bind both polysaccharides (e.g., cellulose, Psl, and Pel) and extracellular proteins (e.g., amyloid fibers) from the biofilm matrix and has been used as an indirect measure of c-di-GMP (Jones and Wozniak, 2017). As shown in Figure 1, the appearance of the F113 wild-type colony on the CR plate was homogeneous, with a dark red color, and rough inside. By contrast, the *amrZ* and *fleQ* mutants presented less binding to CR dye and smoother borders. The *fleQ* mutant showed a thicker red outer halo than the *amrZ* mutant and the staining was slightly more heterogeneous. Regarding the mutants affected in polysaccharides or the production of extracellular proteins, the mutants in *pgaA*, *alg8, psmE*, and *flp-1* showed a lighter color and smoother borders than the wild-type strain, similar to the phenotype observed in the *amrZ* mutant. As for the mutants in the amyloid proteins Fap, in the case of the *fapB* gene, which encodes a minor component of the fiber, no differences in colony morphology or CR staining were observed compared to the wild-type strain. However, mutations in the *fapC* gene, which encodes the fiber monomer, and the *fapF* gene, which encodes the porin implicated in its transport to the extracellular compartment, resulted in a total lack of CR staining. This result indicates that these amyloid fibers were the primary cause of CR staining in *P. ogarae* F113, a strain that lacks cellulose, the main polysaccharide stained by CR in other pseudomonads. The phenotype of the *papA* mutant was similar to the wild-type strain but retained slightly less dye. No relevant differences were found for mutants in the *mapA* gene when compared to the wild-type strain.

**Figure 2:**
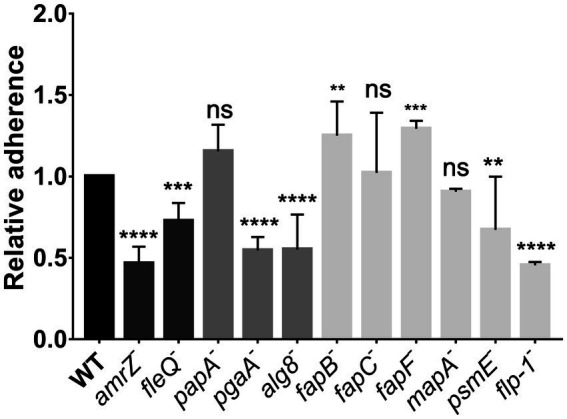
Attachment assays in *P. ogarae* F113 and mutants in extracellular matrix components. Ability of *P. ogarae* F113 wild-type (WT) and derivatives to attach to abiotic surfaces. Crystal violet 2 h-growth biofilms were measured at an OD_590_ and relativized to the WT value. Bars represent average values. Error bars represent the standard deviation obtained from three biological replicas with 16 technical replicates in each experiment. Asterisks denote statistically significant differences according to one-way analysis of variance (ANOVA), with Fisher’s least significant difference (LSD) *post hoc* test (**: *p*-value <0.005, ***: *p*-value <0.0005, ****: *p*-value <0.00005, ns: not significant, *p*-value ≥0.05).

We also analyzed early stages of static biofilm formation, quantified after 2 h-attachment to abiotic surfaces. The 2 hours’ time was chosen in order to show initial attachment and not later stages of biofilm development. The results show that the *amrZ* and *fleQ* mutants were affected (Figure 2). Similarly, the mutants that do not produce PNAG, alginate, the putative adhesin/extracellular epimerase PsmE, or the Flp/Tad pilus were also impaired in the attachment to abiotic surfaces. On the contrary, the *fapF* and *fapB* mutants displayed a slight increase in their attachment/adhesion to abiotic surfaces that were not observed in the *fapC* mutant. The *papA* and *mapA* mutants did not show differences compared to the wild-type strain.

**Figure 3:**
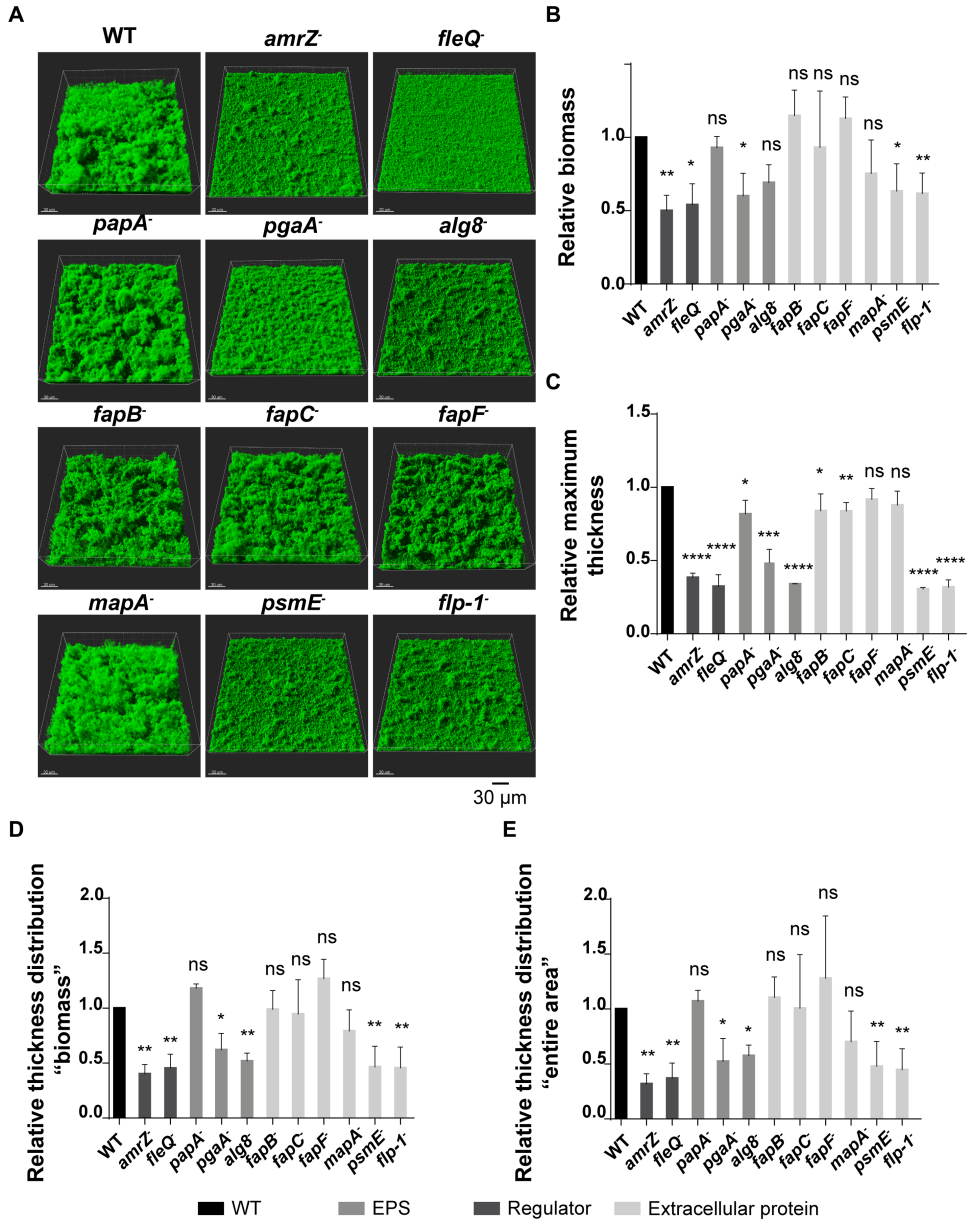
Flow cell experiments in GFP-tagged *P. ogarae* F113 and mutants in extracellular matrix components. **(A)** Confocal laser scanning microscope images of 24 h-old flow-cell biofilms from GFP-tagged wild-type (WT) and derivatives. Images show a 3D projection view. Relative biomass **(B)**, maximum thickness **(C)**, thickness distribution referred to as biomass **(D)** and as entire area **(E)** of biofilms from GFP-tagged F113 derivatives relativized to GFP-tagged wild-type at 24 h. Data represented correspond to four biological replicates with six random images each replicate. Bars represent mean values. Error bars represent the standard deviation. The data was analyzed with COMSTAT2 and unpaired *t*-tests (*: *p*-value <0.05, **: *p*-value <0.005, ***: *p*-value <0.0005, ****: *p*-value <0.00005, ns: not significant, *p*-value ≥0.05).

To further analyze the contribution of ECM components to the biofilm architecture of F113, the wild-type strain and derivatives were tagged with the green fluorescent protein (GFP) to visualize their biofilm formation ability in flow cell reactors, using confocal laser scanning microscopy (CLSM). In these experiments, we analyzed biofilm development during 48 h in flow-cells irrigated with AB medium supplemented with citrate. The 3D-views of CLSM images at 24 h of biofilm development (Figure 3A) showed that all the tested mutants can attach and form biofilms. The wild-type strain built thick and hard biofilms, forming tower-like structures, as previously described (Barahona et al., 2010). On the contrary, biofilm formation was altered in the *amrZ*, *fleQ*, *pgaA*, *alg8*, *psmE*, and *flp-1* mutants, as the tower-like structures were absent, and the biofilms had a flat appearance. All the observed phenotypes remained unaltered after 48 h (Supplementary Figure S1). Biofilms were quantified in terms of biomass (Figure 3B), maximum thickness (Figure 3C), and thickness distribution both in the biomass (Figure 3D) and in the entire area parameters (Figure 3E). At 24 h, the *amrZ, fleQ, pgaA*, *psmE*, and *flp-1* mutants displayed significant lower biofilm biomass and, thickness than the wild-type biofilm. On the other hand, the *alg8* mutant, although presenting a biomass similar to the wild-type strain, was impaired in all the other biofilm parameters. The *mapA*, *fapB*, *fapC*, and *fapF* mutants displayed a biofilm phenotype similar to the wild-type strain. Interestingly, the *papA* mutant was only affected in maximum thickness compared to the wild-type but not in biomass or thickness distribution.

**Figure 4:**
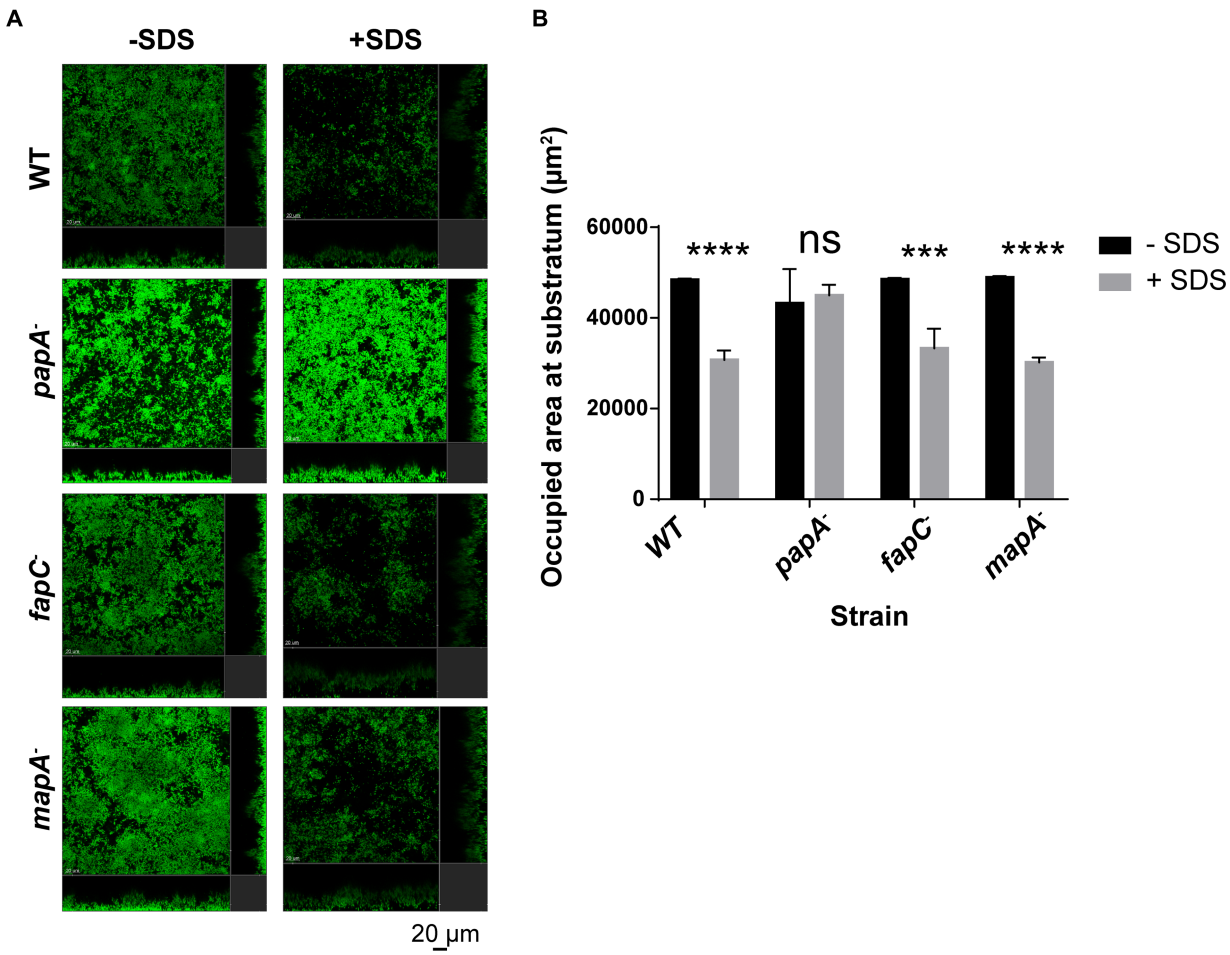
Biofilm stability assay in flow cell devices with GFP-tagged *P. ogarae* F113 and mutants in extracellular matrix components. **(A)** Confocal laser scanning microscope images of 24 h-old flow-cell biofilms formed by the wild-type (WT) strain, *papA*, *fapC*, and *mapA* mutants before and after 40 min of 0.03% sodium dodecyl sulfate (SDS) treatment. A representative image for F113 wild-type and derivatives is shown. Images depict orthogonal sections in the same position before and after treatment. **(B)** Occupied area at the substratum (μm^2^) before and after 40 min of 0.03% SDS treatment from the GFP-tagged WT and derivatives. The experiment was repeated twice. The graph includes the data obtained for a representative experiment with three independent channels per strain. At least four images from independent channel replicates were analyzed for each strain and condition. Bars represent mean values. Error bars represent the standard deviation. The data was analyzed with COMSTAT2 and *t*-tests without correction for multiple comparisons (***: *p*-value <0.0005, ****: *p*-value <0.00005, ns: not significant, *p*-value ≥0.05) to check for statistical differences before and after SDS-treatment in F113 wild-type and derivatives.

All the obtained results show that the disruption of the regulators AmrZ and FleQ, certain polysaccharides as PNAG and alginate, and the extracellular proteins PsmE or Flp/Tad pili led to a dramatic reduction or impairment in biofilm formation. These findings highlight the complexity of biofilm development. Mutants that showed biofilm structures similar to the wild-type strain (*papA^−^*, *fapC^−^* and *mapA^−^*) were further analyzed by evaluating their biofilm stability in flow-cells after treatment with 0.03% of the detergent sodium dodecyl sulfate (SDS). As shown in Figure 4A, the SDS treatment caused a biomass reduction in all tested strains, except in the case of the *papA* mutant. This mutant showed higher resistance to the detergent and more cells remained attached to the substratum, resulting in a higher area occupancy than the other mutants and the wild-type strain (Figure 4B). These results indicate that the Pap polysaccharide also plays a role in biofilm formation and stabilization.

### 3.2 Alginate production decreases swimming motility in *Pseudomonas ogarae* F113

**Figure 5:**
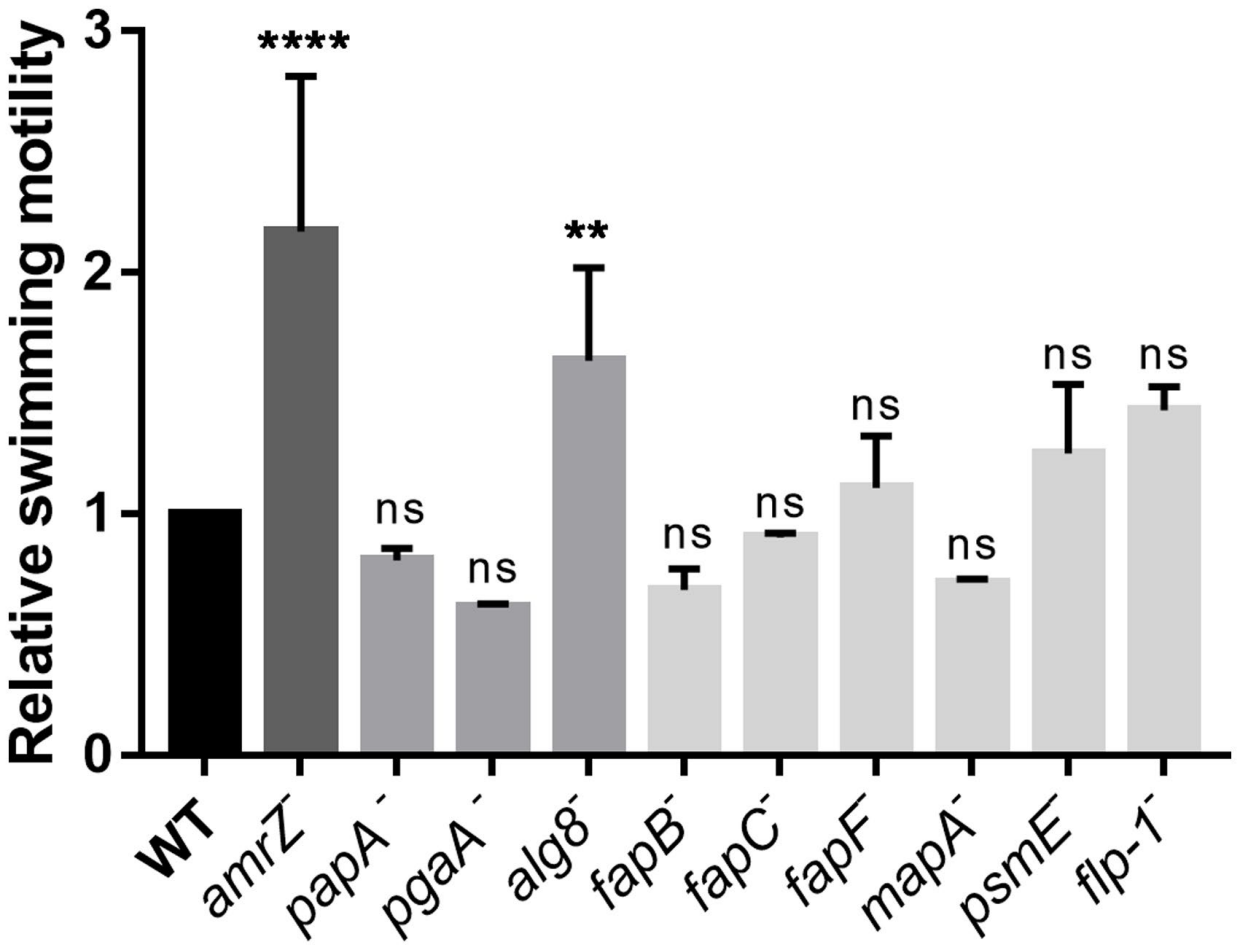
Analysis of the swimming motility ability of *P. ogarae* F113 and mutants affected in the production of extracellular matrix components. The diameter of swimming motility haloes from F113 wild-type (WT) and derivatives was measured at 24 h and relativized to the wild-type strain. The averages of at least three independent biological replicates with three technical replicates (bars), and the standard deviation (error bars) are shown. Asterisks denote statistically significant differences according to one-way ANOVA Fisher’s LSD test (****: *p*-value <0.0001; **: *p*-value <0.01, ns: not significant, *p*-value ≥0.05).

Swimming assays were performed to analyze the influence of the ECM components in the swimming motility of *P. ogarae* F113. In Figure 5, the swimming motility capability of the *amrZ* mutant and the mutants affected in ECM production is shown, relative to the wild-type strain. The *fleQ* mutant was not included as we have previously shown that it is non-motile (Blanco-Romero et al., 2018) due to the lack of flagella. Only the *alg8* mutant displayed a statistically significant increase in motility compared with the wild-type strain, although the hypermotile phenotype was not as important as the previously observed for the *amrZ* mutant (Muriel et al., 2018).

### 3.3 Polysaccharides, extracellular proteins and proteinaceous structures are important ECM components for *Pseudomonas ogarae* F113 competitive rhizosphere colonization

**Figure 6:**
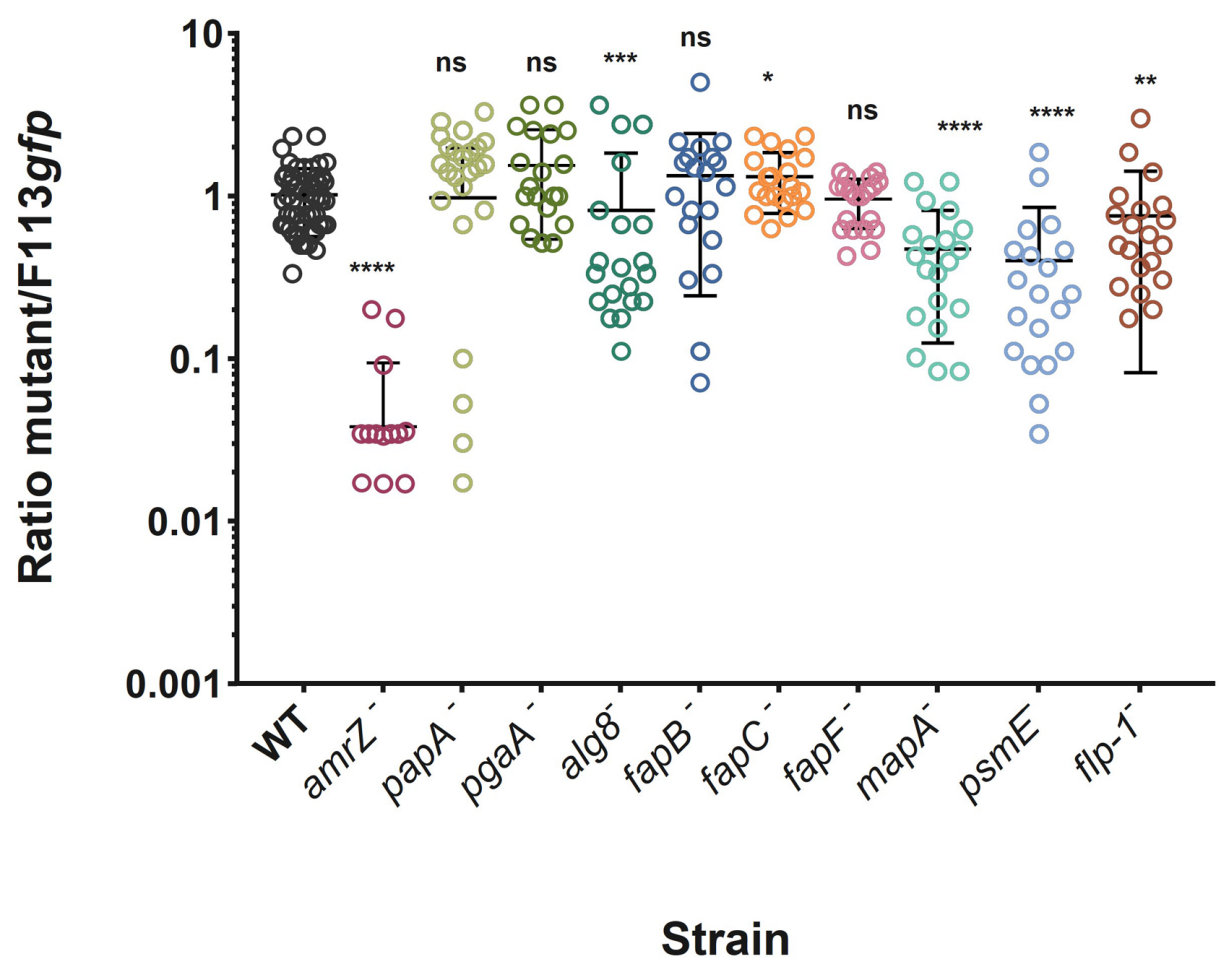
Alfalfa rhizosphere competitive colonization of *P. ogarae* F113 and derivatives affected in the production of extracellular matrix component. *P. ogarae* F113 wild-type (WT) and derivatives were assayed in rhizosphere competitive colonization experiments with alfalfa plants. Each strain was challenged against an F113-*gfp* tagged strain as the competitor, inoculated at a 1:1 ratio. The graph shows the ratio of each strain to F113-*gfp* CFU recovered from the rhizosphere 7 days post-inoculation. Each dot represents the ratio of CFUs recovered from an individual plant. Experiments were repeated at least twice independently with ten independent plants per experiment. Error bars show the standard error of the mean. Asterisks indicate significant statistical differences assessed using Mann–Whitney tests (****: *p*-value <0.0001; ***: *p*-value <0.001; **: *p*-value <0.01; *: *p*-value <0.05; ns: not significant, *p*-value ≥0.05).

To test the involvement of each ECM component in competitive colonization of the rhizosphere by F113, we carried out competition experiments in the rhizosphere of alfalfa seedlings using F113-*gfp* as the competitor. The *fleQ* mutant was not included as its lack of competitivity has already been shown (Capdevila et al., 2004). The results show that the *amrZ* mutant is displaced by the wild-type strain by more than an order of magnitude (Figure 6), as previously reported (Muriel et al., 2018). Furthermore, mutants affected in the production of alginate and the extracellular proteins MapA, PsmE, and Flp/Tad are defective in rhizosphere competitive colonization, although the phenotypes observed were not as dramatic as when *amrZ*^−^ is competing (Figure 6). On the contrary, the *fapC* mutant shows a slight but significant improvement in competitive colonization ability compared to the wild-type strain. No significant differences in colonization were found between the wild-type and the other mutants.

## 4 Discussion

Bacteria utilize diverse mechanisms for surface attachment and biofilm formation, which can vary significantly among different bacterial species. To investigate the contribution of different ECM components to the adaptation of the model rhizosphere bacterium *P. ogarae* F113 to its environment, we constructed mutants in order to avoid each ECM component expression. We have carried out phenotypic analyses that include colony morphology and dye-binding ability assays, the attachment and maturation steps in biofilm formation, motility, and competitive rhizosphere colonization. Mutants in the global TFs AmrZ and FleQ were also included in this study as phenotypic controls and due to their important role in the global regulation of environmental adaptation (Martinez-Granero et al., 2014; Blanco-Romero et al., 2018; Muriel et al., 2018). These two TFs control gene expression of ECM components, both under laboratory culture conditions (Blanco-Romero et al., 2022b) and in the rhizosphere environment (Blanco-Romero et al., 2022a).

Polysaccharides are major components of ECMs and biofilms. *P. ogarae* F113 does not produce cellulose, Psl, Pep or Pea, major components of the ECMs and biofilms formed by other pseudomonads (Blanco-Romero et al., 2020). On the contrary, F113 produces the Pap polysaccharide that is only present in some plant-associated pseudomonads (Blanco-Romero et al., 2020). Like other pseudomonads, F113 also produces, PNAG, a polysaccharide that has been proposed to be substitute for cellulose in biofilm formation of bacteria lacking cellulose production (Lind et al., 2017; Lind, 2019), likely because of its structural similarity. In the case of F113, PNAG appears to be a major biofilm-contributing polysaccharide as determined by the severity of mutant phenotypes in initial attachment and biofilm formation determined by flow cells experiments (Figures 2, 3). In fact, PNAG is a major component of biofilms formed by many Gram-negative and Gram-positive bacteria (Eddenden et al., 2020). In many of these bacteria, PNAG has been shown to be implicated in biofilm formation and host-bacteria interactions (Chen et al., 2014). In different species of *Staphylococcus*, PNAG has been associated with catheter colonization (Burgui et al., 2018) and infectivity (Nguyen et al., 2020). In *Vibrio parahemolyticus*, PNAG has also been implicated in infection (Ye et al., 2014). However, in *P. ogarae* F113, we have not observed a role of PNAG in competitive rhizosphere colonization (Figure 6). In our study we have seen a difference in CR staining indicating that although amyloid fibers are the main target of CR, PNAG can also bind this dye as PNAG is structurally similar to cellulose, with b-1,6 bonds.

The other polysaccharide that contributed to biofilm formation is alginate (Figures 2, 3). Alginate is a co-polymer of D-mannuronic and L-guluronic acids that is widely present in the *Pseudomona*s genus (Blanco-Romero et al., 2020). Its production has been extensively studied in *P. aeruginosa* (Hentzer et al., 2001; Nivens et al., 2001; Wozniak et al., 2003; Tielen et al., 2005) and *P. syringae* (Keith et al., 2003; Laue et al., 2006; Helmann et al., 2019), playing a role in biofilm structure in abiotic surfaces, cellular aggregation during microcolony formation, epiphytic fitness, virulence, protection against antibiotics and the human immune system (Hentzer et al., 2001; Nivens et al., 2001; Pier et al., 2001; Leid et al., 2005; Tielen et al., 2005; Laue et al., 2006; Helmann et al., 2019). Similarly, in *P. putida*, alginate is required under water-stress conditions to maintain a proper biofilm structure (Chang et al., 2007). However, in *P. chlororaphis* PCL1606, alginate only plays a secondary role during biofilm formation, conferring a competitive advantage during biofilm formation in cooperation with other extracellular components (Heredia-Ponce et al., 2020b). Marshall et al. showed that mutants of *P. fluorescens* Pf0-1 affected in alginate production were impaired in the colonization of both moist and dry soils (Marshall et al., 2019). As it was previously shown that *alg8* knockouts in *P. aeruginosa* led to complete loss of alginate production (Remminghorst and Rehm, 2006), we proceeded to construct a mutant affected in *alg8* in F113. Besides its role in biofilm formation, this mutant shows a different morphology and dye-binding pattern (Figure 1) than the wild-type strain and a hypermotile phenotype (Figure 5). Differences in swimming motility are probably due to an increase in media viscosity by the alginate producing strain. Lower surface coverage and a flattened structure were also observed in a *P. syringae alg8* mutant compared to the wild-type strain (Heredia-Ponce et al., 2020a). Although the crucial role of alginate in the biofilm development process in other pseudomonads has been demonstrated mainly in relation to harsh environments and *in planta*, soil, or lungs (Wozniak et al., 2003; Chang et al., 2007; Helmann et al., 2019; Marshall et al., 2019; Heredia-Ponce et al., 2020a,b); in the case of the *P. ogarae* F113, it seems also to play a predominant role in the process of biofilm formation on abiotic surfaces (Figures 2, 3). In addition, alginate in *P. ogarae* F113 is also important for the rhizosphere fitness, being displaced by the wild-type strain in rhizosphere competitive colonization assays (Figure 6).

Another polysaccharide putatively present in the *P. ogarae* ECM is Pap (Blanco-Romero et al., 2020). While the *papA* mutant showed no impact on initial attachment or biofilm structure, it appeared to play a role in biofilm stability (Figure 4). This inference arises from the distinct resistance to detergent treatment observed in the *papA* mutant (Figure 4). To our knowledge, the implication of this polysaccharide in biofilm formation has not been reported before.

Besides polysaccharides, it is important to note that in F113 two extracellular proteins, PsmE and the Flp/Tadb pili, have an important impact in biofilm formation as its mutants are affected in attachment and biofilm formation (Figures 2, 3). Moreover, in the flow cell experiments made with the *psmE^−^* and *flp/tad^−^* mutants, there is a clear decrease in both, biofilm biomass and entire occupied area (Figures 3C–E). The PsmE protein was originally described in *Pseudomonas syringae* as a RTX protein with a bifunctional enzymatic activity (Bjerkan et al., 2004). This protein contains two enzymatic domains and two calcium binding domains. The enzymatic domains had mannunoran epimerase and acetyl hydroxylase activities. Alginate was a natural substrate for both enzymatic activities, since PsmE was able to deacetylate mannunoran residues of this polysaccharide, that were subsequently epimerized to glucuronan. Glucuronation of alginate resulted in an increase of gelification properties of alginate. The F113 PsmE orthologue also has the same domains and shows high sequence homology, indicating a similar role in this bacterium. Based on the known mechanism of action of PsmE in other pseudomonads, it is plausible that in F113 PsmE functions to modify alginate and increase its gelification properties, thereby contributing to the overall architecture of the biofilm. In addition, it is interesting to note that the Pap polysaccharide contains mannunoran residues (Blanco-Romero et al., 2020) and could therefore be a substrate for PsmE too. The lack of alginate modification could explain the impairment of the *psmE* mutant in rhizosphere competitive colonization experiments, similarly to the *alg8* mutant. To our knowledge, PsmE has not been linked before with biofilm formation or with host colonization.

On the other hand, the role of the Flp/tad pilus, another extracellular protein involved in biofilm formation, has been described within the *Pseudomonas* genus with respect to its function in motility, biofilm formation, and colonization (Lugtenberg et al., 2001; Cole et al., 2017). There are two configurations of the Flp/Tad pilus in the *Pseudomonas* genus: type A, described for *P. aeruginosa* (de Bentzmann et al., 2006), which is widely distributed in the genus, and the newly described type B, which is restricted to the *Pseudomonas fluorescens* complex (Blanco-Romero et al., 2020). However, the specific role of both type A and B Flp/Tad pili in pseudomonads remains unexplored. Here we found that the disruption of the Flp/Tad type B pilus formation in F113 is important not only for biofilm formation, but also for the bacterial adaptation to the rhizosphere environment, since the *flp-1* mutant is impaired in rhizosphere competitive colonization (Figure 6).

The first step in biofilm formation is the attachment of cells to surfaces, which in the *Pseudomonas fluorescens* complex has been demonstrated to be predominantly achieved through different type I-secreted RTX adhesion proteins. The LapA adhesin is involved in the initial bacterial attachment in *P. putida* and *P. fluorescens* and its role in biofilm formation has been elucidated (Hinsa et al., 2003; Newell et al., 2009, 2011; Ivanov et al., 2012). In addition, there are other adhesins such as LapF in *P. putida* (Espinosa-Urgel et al., 2000; Fuqua, 2010; Martínez-Gil et al., 2010, 2012, 2013) and MapA in *P. fluorescens*, which also participate in cohesion under specific conditions (Collins et al., 2020). Nevertheless, our findings suggest that MapA does not contribute to F113 biofilm formation, at least under the tested conditions. Collins et al. (2020) found that *mapA* is only expressed in the thicker regions of mature biofilms where oxygen and nutrients are scarce. Indeed, they were only able to observe a distinctive biofilm-related phenotype for the *P. fluorescens* Pf0-1 *mapA* mutant in a specific medium (K10-T supplemented with L- arginine) that leads to high levels of c-di-GMP. It is interesting to note that although it seems that this adhesin does not have a role in biofilm formation in F113, it is important during rhizosphere competitive colonization (Figure 6), suggesting a role in the F113 fitness during the interaction with plants. This finding agrees with the already described role of adhesins during rhizosphere colonization (Espinosa-Urgel et al., 2000; Hinsa et al., 2003; Yousef-Coronado et al., 2008).

In recent years, additional proteinaceous structures have been identified as being associated with biofilm formation and niche or host colonization. Among these are the Fap proteins, which are secreted proteins capable of self-assembling into β-sheet-rich fibrillar aggregates (Dueholm et al., 2010). In *P. aeruginosa*, Fap proteins confer mechanical strength to the extracellular matrix during biofilm formation (Dueholm et al., 2010, 2013; Zeng et al., 2015) and play a role in virulence (Wiehlmann et al., 2007). Recently, Fap has been associated with biofilm formation in the plant growth-promoting rhizobacterium *P. chlororaphis* PCL1606. While Fap is not essential for the biofilm architecture, it promotes bacterial competition during biofilm formation, facilitates early attachment to surfaces, and contribute to the biocontrol activity of the bacterium (Heredia-Ponce et al., 2020b). In the case of *P. fluorescens* PF07 Fap seems essential for biofilm formation as a mutant in the regulator of Fap synthesis is defective in forming mature microcolony biofilms (Guo et al., 2022). For the study of Fap in *P. ogarae* F113, *fapB, fapC*, and *fapF* mutants were constructed. The most noticeable differences were found concerning morphology and dye-binding (Figure 1), due to the capacity of CR to non-specifically stain amyloids (Jones and Wozniak, 2017). According to our results, amyloid proteins are the main responsible of CR staining in F113. The staining of the *fapC* and *fapF* mutants, affected in the major component and the secretion pore of Fap, respectively, is very reduced compared to the wild-type, which agrees with earlier observations in *P. fluorescens* UK4 (Zeng et al., 2015). However, staining of the *fapB* mutant was not substantially modified, revealing its secondary role in the fibers, as previously reported (Rouse et al., 2018). Furthermore, an increase in the attachment ability (Figure 2) and rhizosphere competitive colonization (Figure 6) was observed for *fapB* and *fapF*, and *fapC* mutants, respectively, compared to the wild-type strain, suggesting that the production of these amyloid proteins can be detrimental for F113 under the tested conditions or could play a role in recognition of the bacteria by the plant defense. In this sense, Kosolapova et al. (2019) described that functional amyloids could play a role in *R. leguminosarum* plant symbiosis based on the higher expression of genes encoding the amyloid when they add plant flavonoids mimicking the initial steps of nodulation (Tolin et al., 2013; Kosolapova et al., 2019).

## 5 Conclusion

The phenotypic characterization of mutants affected in the production of the major EMC components in *P. ogarae* F113 has demonstrated their role in biofilm formation and adaptation of this bacterium to the rhizosphere environment. Together, the results presented here show that the polysaccharides PNAG and alginate, the extracellular protein PsmE, and the Flp/Tad type B pilus are the major biofilm-supporting matrix components in this bacterium. Furthermore, we have also demonstrated that alginate, PsmE, Flp/Tad, and the recently described adhesin MapA are required for effective rhizosphere competitive colonization.

## Data availability statement

The original contributions presented in the study are included in the article/Supplementary material, further inquiries can be directed to the corresponding author.

## Author contributions

EB-R: Formal analysis, Investigation, Methodology, Writing – original draft, Writing – review & editing. DG-S: Formal analysis, Investigation, Methodology, Writing – review & editing. DD: Formal analysis, Investigation, Methodology, Writing – review & editing. MR: Investigation, Methodology, Validation, Writing – review & editing. TT-N: Conceptualization, Supervision, Validation, Writing – review & editing. MR-N: Formal analysis, Investigation, Methodology, Validation, Writing – review & editing. RR: Conceptualization, Funding acquisition, Supervision, Validation, Writing – original draft, Writing – review & editing. MM: Conceptualization, Funding acquisition, Supervision, Validation, Writing – original draft, Writing – review & editing.
