# Supplementary material for: Role of extracellular matrix components in biofilm formation and adaptation of Pseudomonas ogarae F113 to the rhizosphere environment
Source: Front Microbiol. 2024 Jan 25;15:1341728. doi: 10.3389/fmicb.2024.1341728 (PMC10850567; doi:10.3389/fmicb.2024.1341728)
Supplement: Supplementary file 1 [file Data_Sheet_1.PDF]

Supplementary Material

Role of extracellular matrix components in biofilm formation and adaption of *Pseudomonas ogarae* F113 to the rhizosphere environment

Esther Blanco-Romero[1], Daniel Garrido-Sanz[1,2], David Durán[1], Morten Rybtke[3], Tim Tolker-Nielsen[3], Miguel Redondo-Nieto[1], Rafael Rivilla[1] and Marta Martín[1*]

[1] Departamento de Biología, Facultad de Ciencias, Universidad Autónoma de Madrid, Darwin 2, 28049 Madrid, Spain.

[2] Department of Fundamental Microbiology, University of Lausanne, 1015 Lausanne, Switzerland.

[3] Costerton Biofilm Center, Department of Immunology and Microbiology, University of Copenhagen, Blegdamsvej 3b, 2200 Copenhagen N, Denmark

*** Correspondence:**
Marta Martin, m.martin@uam.es

Supplementary Table S1 Bacterial strains and plasmids used in this work.

**Strains Plasmids**	**Description**	**Reference**
P. ogarae		
F113	*P. ogarae* F113 wild type, Rif^R	(Shanahan et al., 1992)
amrZ$^-$	*P. ogarae* F113 *amrZ*$^-$, Rif^R, Km^R	(Martínez-Granero et al., 2012)
fleQ$^-$	*P. ogarae* F113 *fleQ*$^-$, Rif^R, Km^R	(Blanco-Romero et al., 2018)
papA$^-$	*P. ogarae* F113 *papA*$^-$, Rif^R, Km^R	This work
pgaA$^-$	*P. ogarae* F113 *pgaA*$^-$, Rif^R, Km^R	This work
alg8$^-$	*P. ogarae* F113 *alg8*$^-$, Rif^R, Km^R	This work
fapB$^-$	*P. ogarae* F113 *fapB*$^-$, Rif^R, Km^R	This work
fapC$^-$	*P. ogarae* F113 *fapC*$^-$, Rif^R, Km^R	This work
fapF$^-$	*P. ogarae* F113 *fapF*$^-$, Rif^R, Km^R	This work
mapA$^-$	*P. ogarae*F113 *mapA*$^-$, Rif^R, Km^R	This work
psmE$^-$	*P. ogarae* F113 *psmE*$^-$, Rif^R, Km^R	This work
flp1$^-$	*P. ogarae* F113 *flp-1*$^-$, Rif^R, Km^R	This work
F113 gfp	*P. ogarae* F113 wild type, tagged with miniTn7-*gfp-gm*, Rif^R, Gm^R	This work
amrZ$^-$ gfp	*P. ogarae* F113 *amrZ*$^-$, tagged with miniTn7-*gfp-gm,* Rif^R, Km^R, Gm^R	This work
fleQ$^-$ gfp	*P. ogarae* F113 *fleQ*$^-$, tagged with miniTn7-*gfp-gm,* Rif^R, Km^R, Gm^R	This work
papA$^-$ gfp	*P.ogarae* F113 *papA*$^-$, tagged with miniTn7-*gfp-gm,* Rif^R, Km^R, Gm^R	This work
pgaA$^-$ gfp	*P. ogarae* F113 *pgaA*$^-$, tagged with miniTn7-*gfp-gm,* Rif^R, Km^R, Gm^R	This work
alg8$^-$ gfp	*P. ogarae* F113 *alg8*$^-$, tagged with miniTn7-*gfp-gm,* Rif^R, Km^R, Gm^R	This work
fapB$^-$ gfp	*P. ogarae* F113 *fapB*$^-$, tagged with miniTn7-*gfp-gm,* Rif^R, Km^R, Gm^R	This work
fapC$^-$ gfp	*P. ogarae* F113 *fapC*$^-$, tagged with miniTn7-*gfp-gm,* Rif^R, Km^R, Gm^R	This work
fapF$^-$ gfp	*P. ogarae* F113 *fapF*$^-$, tagged with miniTn7-*gfp-gm,* Rif^R, Km^R, Gm^R	This work
mapA$^-$ gfp	*P.ogarae* F113 *mapA*$^-$, tagged with miniTn7-*gfp-gm,* Rif^R, Km^R, Gm^R	This work
psmE$^-$ gfp	*P. ogarae* F113 *psmE*$^-$, tagged with miniTn7-*gfp-gm,* Rif^R, Km^R, Gm^R	This work
flp1$^-$ gfp	*P. ogarae* F113 *flp-1*$^-$, tagged with miniTn7-*gfp-gm,* Rif^R, Km^R, Gm^R	This work

Supplementary Table S1 continuation

Strains<br>Plasmids	Description	Reference
E. coli		
DH5α	*E. coli* cloning strain, φ80*lacZ*ΔM15, Δ(*lacZYA-argF*), U169, *recA1*, *endA1*, *hsd*R17 (r_K^-, m_K^+), *phoA*, *sup*E44-λ-*thi*-1, *gyrA96*, *relA1*	Gibco, BRL
**Plasmids**		
miniTn7-*gfp-gm*	Source of miniTn7-*gfp-gm* transposon for Gfp-tagging, Gm^R, Ap^R	(Koch et al., 2001)
pUX-BF13	Helper plasmid providing the Tn7 transposition functions in trans, Ap^R, mob^+, ori-R6K	(Bao et al., 1991)
pRK600	Helper plasmid, Cm^R	(Finan et al., 1986)
pCR™2.1TOPO™	Cloning vector, Ap^R, Km^R	Invitrogen
pCR™2.1TOPO™-	Cloning vector with an *Eco*RI fragment of *papA* gene, Ap^R, Km^R	This work
pCR™2.1TOPO™-	Cloning vector with an *Eco*RI fragment of *pgaA* gene, Ap^R, Km^R	This work
pCR™2.1TOPO™-	Cloning vector with an *Eco*RI fragment of *alg8* gene, Ap^R, Km^R	This work
pCR™2.1TOPO™-	Cloning vector with an *Eco*RI fragment of *fapB* gene, Ap^R, Km^R	This work
pCR™2.1TOPO™-	Cloning vector with an *Eco*RI fragment of *fapC* gene, Ap^R, Km^R	This work
pCR™2.1TOPO™-	Cloning vector with an *Eco*RI fragment of *fapF* gene, Ap^R, Km^R	This work
pCR™2.1TOPO™-	Cloning vector with an *Eco*RI fragment of *mapA* gene, Ap^R, Km^R	This work
pCR™2.1TOPO™-	Cloning vector with an *Eco*RI fragment of *psmE* gene, Ap^R, Km^R	This work
pCR™2.1TOPO™-	Cloning vector with an *Eco*RI fragment of *flp-1* gene, Ap^R, Km^R	This work

References

Bao, Y., Lies, D.P., Fu, H., and Roberts, G.P. (1991). An improved Tn7-based system for the single-copy insertion of cloned genes into chromosomes of gram-negative bacteria. *Gene* 109(1)**,** 167-168.

Blanco-Romero, E., Redondo-Nieto, M., Martínez-Granero, F., Garrido-Sanz, D., Ramos-González, M.I., Martín, M., et al. (2018). Genome-wide analysis of the FleQ direct regulon in *Pseudomonas fluorescens* F113 and *Pseudomonas putida* KT2440. *Sci Rep* 8(1)**,** 13145.

Finan, T.M., Kunkel, B., De Vos, G.F., and Signer, E.R. (1986). Second symbiotic megaplasmid in Rhizobium meliloti carrying exopolysaccharide and thiamine synthesis genes. *Journal of bacteriology* 167(1)**,** 66-72.

Koch, B., Jensen, L.E., and Nybroe, O. (2001). A panel of Tn7-based vectors for insertion of the *gfp* marker gene or for delivery of cloned DNA into Gram-negative bacteria at a neutral chromosomal site. *J Microbiol Methods* 45(3)**,** 187-195.

Martínez-Granero, F., Navazo, A., Barahona, E., Redondo-Nieto, M., Rivilla, R., and Martín, M. (2012). The Gac-Rsm and SadB signal transduction pathways converge on AlgU to downregulate motility in *Pseudomonas fluorescens*. *PLoS One* 7(2)**,** e31765.

Shanahan, P., O'Sullivan, D.J., Simpson, P., Glennon, J.D., and O'Gara, F. (1992). Isolation of 2, 4-diacetylphloroglucinol from a fluorescent pseudomonad and investigation of physiological parameters influencing its production. *Appl. Environ. Microbiol.* 58(1)**,** 353-358.

Supplementary Table S2 Primers used in this work

Name	Sequence 5'-3'	Description
pgaA_F	GTTGATCGACCGTGGCGTG	For amplification of a *pgaA* (PSF113_0161) fragment for mutation by insertional inactivation
pgaA_R	CCGAACAGCCGCCAGTCTTC	
papA_F	GCGTATTTGGTACGGGGTATGTCGG	For amplification of a *papA* (PSF113_1970) fragment for mutation by insertional inactivation
papA_R	GCTTCCATCAGGTAGCGGCTG	
mapA_F	GCCTCGGTGGTGGAGGGCG	For amplification of a *mapA* (PSF113_1511) fragment for mutation by insertional inactivation
mapA_R	AAGTACGTCGTTGTCCAGC	
psmE_F	CGTGGTACCGGCCACCAG	For amplification of a *psmE* (PSF113_3004) fragment for mutation by insertional inactivation
psmE_R	GGGCGCTGCCGTTGGCGAG	
fapB_F	CCTGCAGGACTCTGGCAG	For amplification of a *fapB* (PSF113_2684) fragment for mutation by insertional inactivation
fapB_R	GGTAGCAACGCCACGTTCTG	
fapC_F	CTCAATGGCAGCAACGGGAAC	For amplification of a *fapC* (PSF113_2683) fragment for mutation by insertional inactivation
fapC_R	CAACGGTGCCGGTGCCTTTG	
fapF_F	GTTTCGAGACTGGCGTGACC	For amplification of a *fapF* (PSF113_2680) fragment for mutation by insertional inactivation
fapF_R	AAAGCCCGGGCGTGATCG	
flp1-F	CTTCGGTACTGAAGTTCGCCAAA	For amplification of an *flp-1* (PSF113_4192) fragment for mutation by insertional inactivation
flp1-R	TGGTATTCACGGCGCC	
alg8-F	CAAGTGTATGGCTCGGTGATT	For amplification of an *alg8* (PSF113_4753) fragment for mutation by insertional inactivation
alg8-R	GAACCAGCTCGATTTGTCATC	

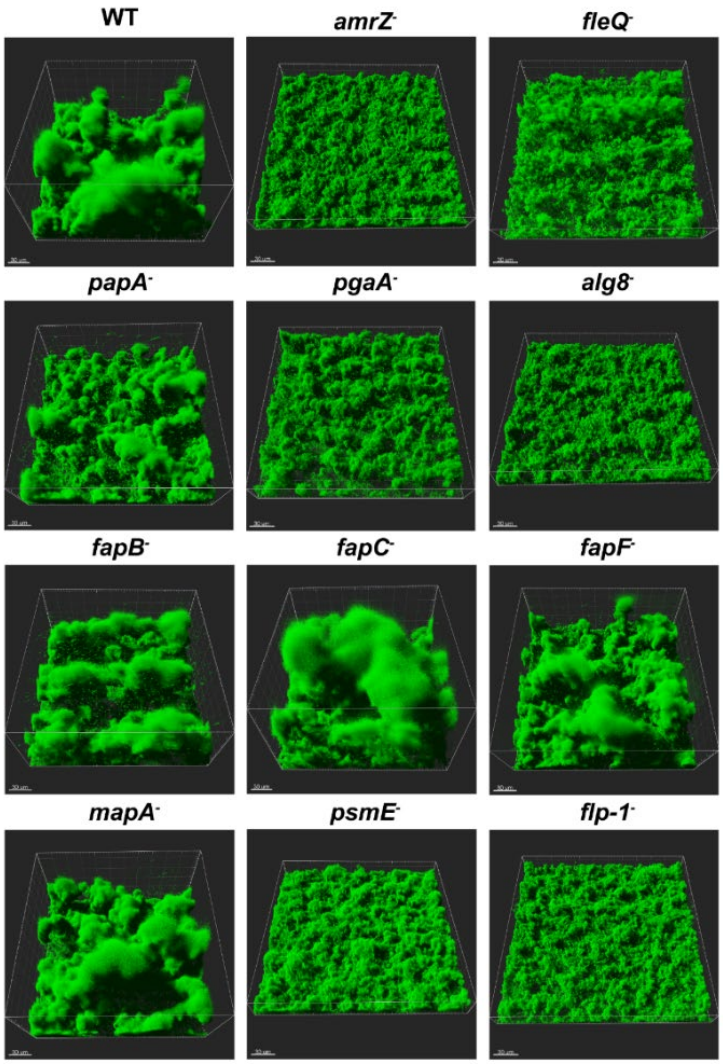

Supplementary Figure S1: 48 h flow cell experiments in GFP-tagged *P. ogarae* F113 and mutants in ECM components. Confocal laser scannin g microscope (CLSM) images of 48 h-old flow cell biofilms from GFP-tagged wild type and derivatives (*amrZ*, *fleQ*, *papA*, *pgaA*, *alg8*, *fapB*, *fapC*, *fapF*, *mapA*, *psmE,* and *flp-1* mutants). Images show the surface rendering of 3D volume projection views. Scale bars depict 30 um.
